# Opportunities for reproductive tourism: cost and quality advantages of Turkey in the provision of in-vitro Fertilization (IVF) services

**DOI:** 10.1186/s12913-016-1628-7

**Published:** 2016-08-12

**Authors:** M. Said Yildiz, M. Mahmud Khan

**Affiliations:** 1 Ministry of Health, Ankara, Turkey; 2 Department of Health Services Policy and Management, University of South Carolina, 915 Greene Street, #357, Columbia, SC 29208 USA; 3 Department of Health Services Administration, China Medical University, Taichung City, Taiwan

**Keywords:** Medical tourism, In-vitro fertilization, Tourism destination, Cost of IVF services, Quality of health care

## Abstract

**Background:**

The scale and scope of medical tourism have expanded rapidly over the last few decades. Turkey is becoming an important player in this market because of its relatively better service quality and large comparative cost advantage.

**Methods:**

This paper compares cost, quality and effectiveness of in-vitro fertilization (IVF) in the USA and in Turkey. The data from Turkey were obtained from a hospital specializing in IVF services and the US data came from secondary sources. Package price offered by the dominant IVF-service provider to international patients in Turkey was used as a measure of cost for Turkey while IVF-specific service prices were used to estimate the cost for USA. To compare quality and effectiveness of IVF services, a number of general clinical quality indicators and IVF success rate were used.

**Results:**

Indicators of quality, cost and success rate in the Turkish hospital were found to be better than the corresponding indicators in US hospitals. The cost difference of IVF services between USA and Turkey is so significant that the overall cost of obtaining the service from Turkey remains lower even with additional expenses for travel and accommodation.

**Conclusions:**

Cost-effectiveness ratio of IVF treatment per successful clinical pregnancy was much lower in Turkey than in the USA. It appears that cost and quality are the two most important factors affecting demand for health care services by international patients in Turkey. Like other important players in the medical tourism market, Turkey should be able to take advantage of its success in IVF, a highly specialized niche market, to transform its health system into an important exporter of general health services.

## Key messages

Medical tourism market has been expanding rapidly over the last few decades. Policy makers in Turkey consider this an important growth sector and the number of medical tourists arriving Turkey has been increasing at a rapid rate.

Turkey shows significant comparative advantage in the provision of in-vitro fertilization treatment. Cost and quality appear to be the most important factors affecting the demand for medical services internationally. Using the highly specialized medical service as the catalyst, Turkey should be able to become an important player in medical tourism industry.

## Background

With the increase in international movement of capital, inputs of production, consumer goods, and services, global trade in health commodities and services has grown rapidly. The nature and composition of international trade in health services is undergoing rapid transformations with technological developments in medicine and communications. Although flow of medical commodities was always important in international trade, in recent years movement of patients across borders has become increasingly significant. With increasing demand for medical care internationally, a number of countries have identified medical tourism as an important growth sector. For example, countries like Thailand, Singapore, India, Turkey, have adopted specific policies to encourage and subsidize international flow of patients. Wider diffusion of modern medical technology is increasing the competitiveness in this market, encouraging product differentiation across the countries. Early adopters of medical tourism (Thailand, Philippines, Singapore, and India) started with specialized services and then branched-out to provide more broad-based services but further expansion of the market is encouraging greater degree of specializations in advanced medical procedures and interventions. Aspiring medical service exporters are also trying to identify medical specializations in which they might have comparative advantage because of their geographic locations, economic structure and quality and efficiency of health care systems.

Medical Tourism takes place when individuals travel to another country with the primary intention of receiving medical treatments and services. There are a number of reasons why patients seek care internationally rather than obtaining the services in their own country of residence. The reasons may include non-availability of services locally, lack of insurance coverage, long waiting time, higher quality and lower cost in the destination countries, possibility of combining medical care with tourism, other factors related to personal taste and preferences. Depending on the type of care needed and the benefits and costs of accessing the services internationally, the trips may be short distance (for example, travel within Europe) or long or even intercontinental. The flow of patients is also not unidirectional from developed to developing countries; patients flow from one developed country to another, from developed to developing regions, between developing areas or from developing areas to developed economies.

The flow of patients to developed countries is not a new phenomenon. Richer and privileged patients from both developed and developing countries sought care in developed countries mainly because of perceived high quality of services. In the past, travelling overseas for medical care from developing countries was limited to high-income, politically connected individuals [1]. Flow of patients between developed countries, mainly within the European Union [2] and between USA and Canada was also common [3]. In recent years, the magnitude and direction of patient flow has changed drastically. Alsharif et al. [4] mentioned Middle Eastern countries like United Arab Emirates and Jordan as important destinations for patients from the region because of their relatively well-developed healthcare infrastructure. Ramirez [5] found similar flow to Mexico, Cuba, Panama and Costa Rica from the neighboring countries. A number of Southeast Asian countries like Thailand, Singapore, Malaysia and India in South Asia, have become important destinations for patients from both developed and developing countries [6].

Range of treatments available overseas include cosmetic and dental surgery; cardio, orthopedic and bariatric surgery; in-vitro fertilization (IVF) and related treatments; and organ transplantation and other general services [7]. Medical travel decisions are affected by many factors like patient characteristics (insured or uninsured, socioeconomic situation, age and disabilities), medical care in the home country and in destination country (quality, cost, ease of access), existence of formal incentive mechanisms (encouraged by employers, insurance agencies, governments), type of medical care treatment being sought (cosmetic, elective, mandatory, emergency, highly specialized procedures, legally not allowed in home country), psychological factors associated with travel and obtaining care from unfamiliar cultural and social context, possibility of unexpected expenses, complications, malpractice, etc. For countries without universal medical insurance like United States, cost savings has been reported as one of the most important factors for seeking care internationally while in countries with national health programs outflows of patients are mainly for obtaining services not covered by the national program [8] or to avoid waiting line [9].

Lower transportation costs, instant electronic communication system, low time costs of travel have enabled many countries to enter the medical tourism market. Turkey is also taking advantage of this global change and has been encouraging foreign patients to seek care in Turkey. As a destination country, Turkey has become quite popular in recent years, probably due to the provision of quality services at a relatively low cost, low waiting time and good reputation of some specialized medical care facilities and professionals. With rapid expansion of medical tourism, competition among potential destination countries has also become more intense. Market competition has encouraged countries to become specialized in a few highly specific medical interventions and services. Although the degree of specialization of Turkish health system is still evolving, in-vitro fertilization has emerged as one of the important areas in which the country appears to have some comparative advantage over other medical care interventions. The purpose of this study is to better understand the factors associated with increased popularity of medical tourism in Turkey through a case study of in-vitro fertilization (IVF) treatment.

### Size of medical tourism market

Despite the fact that international trade in medical services has been growing rapidly over the last few decades, data on medical tourism and number of medical tourists worldwide are not readily available. Although some numbers are referred to, these are not based on any systematic collection of data [10]. Anecdotal evidences are often presented to indicate high rates of growth of medical tourism in India, Thailand and Mexico, but objective information is hard to come by. The Tourism Research and Marketing reported that the global medical tourism market had over 19 million trips in 2005 with a total monetary value of $20 billion. Since a number of countries were experiencing double-digit growth in 2005, the projection was that medical tourism will double in value by 2010 [11]. Deloitte Medical Tourism Report estimated that approximately 750,000 Americans traveled outbound for medical care in 2007. Deloitte projected a 100 % annual rate of growth through 2010 [12]. Among the OECD countries, the largest importer of medical care is Germany with US$1.5 billion in value while the United States is the largest exporter with US$2.3 billion in exports. Among the OECD countries, Turkey has become an important exporter of medical care (export value of US$409 million), third largest exporter after USA and Czech Republic [13]. McKinsey report, on the other hand, estimated that the number of medical tourists from the USA was only 75,000 if service use by expatriates and emergency cases is excluded, asserting that “the market is much smaller than conventional wisdom suggests” [14].

In Turkey, medical tourism is considered an important growth sector and policy makers are eager to help expand this market. Ministry of Health medical tourism department reported that the number of medical tourists arriving Turkey increased from 74,093 in 2005 to 261,999 in 2011 [15]. The Turkish data on medical tourists may also represent significant overestimation. It is interesting that the same report mentioned that the majority of foreign patients in Turkey were the patients who needed emergency care and therefore the number of “real” medical tourism patients (those who travel to Turkey primarily for seeking medical treatment) should be much smaller. It is also possible that patients misrepresent their medical care use as emergency type in order to be able to receive reimbursements from their home-country insurance agencies.

### Specialization in medical tourism

As indicated above, with increasing competition in the medical tourism market, countries are trying to define their own areas of specializations to create differentiation and market segmentation. Countries tend to specialize in market niches on the basis of the resources they have, medical care infrastructure of the countries, levels of development of tourism and trade opportunities. Although some countries may decide to focus on general medical care services, differentiation allows countries to develop some market power in a highly competitive environment. The differentiations are often created by offering specialized medical care services, the services in which the countries show significant comparative advantage. Investments in these narrowly defined specializations allow the countries to become well-known as the preferred destination for those specific services.

Becoming recognized as the preferred destination for highly specialized medical care services creates the “reputation-effect”, which may become the catalyst for achieving higher market demand for other medical services as well. For example, Poland and Hungary started medical tourism with dental treatments because of their significant cost advantage over the neighboring countries [3]. This initial specialization in dental care triggered development in other medical care fields and some of these Eastern European countries became well-known destinations for plastic surgery and other elective treatments. Similarly, Connell [16] reported that Thailand specialized in sex change surgery at the early stage but gradually became important destination for general medical care services, cosmetic surgery, heart surgery, etc. South Africa and Argentina also specialized in cosmetic surgery and attracted patients from their own geographic regions.

A number of countries have taken advantage of their special status as the centers of specific religion to become recognized as medical tourism destinations for people with the religious identity. Inhorn [17] mentioned that Iran and Lebanon have become destination for a significant number of Shiite population. Israel attracts many Jewish people from around the world [18]. Travel for IVF or surrogacy to a number of Asian countries has become popular because of lower regulatory restrictions on surrogacy and sex-selection in addition to the price-advantage these countries offer [19].

Sometimes, focusing on particular treatment stems from destination countries’ location and for being a travel hub for regional countries with relatively low heath care quality. Accredited hospitals in Jordan and Turkey became specialized in state-of-the-art cancer treatments and attracted patients from the neighboring countries. Similarly, Indian hospitals became well-known in the diagnosis and treatment of cardiovascular diseases while Argentina became specialized in eye surgeries. Malta became a destination for hip and knee replacement. The ability of the destination countries in using trained medical professionals, modern technology and scientifically recognized methods has also attracted medical tourists [20]. Once a country becomes well-known in the provision of specific medical services, economies of scale allows it to capture even a higher market share [21]. The ability of countries to package medical services with general tourism improves cost-advantage for patients [22].

Medical tourism facilitators use the country-specific specializations for guiding their customers in the choice of countries and health care facilities. Medical tourism websites emphasize these specializations to encourage international travel for obtaining medical care. For example, medical tourism websites often mention India for orthopedic surgery (especially knee resurfacing) and cardiac surgery; Singapore for cancer treatment, spinal surgery, transplants, and Mexico for dental treatments. India, Thailand, Singapore and Malaysia have also become very successful in attracting long-distance medical tourists from developed countries to their JCI accredited hospitals [23]. These hospitals are comparable to some of the best hospitals in the world [24].

### Medical tourism, In-Vitro Fertilization (IVF) and Turkey as a destination country

In reproductive health area, the IVF technique has remained relatively unchanged over the last 30 years. Treatments start with the stimulation of ovaries in order to trigger production of large number of eggs, some of which can be retrieved. Retrieved eggs are placed in a tube for fertilization and sperm is introduced in the solution to get fertilized eggs. After 2–5 days, the fertilized embryos are transferred into the uterus of the mother-to-be with the help of a catheter [25].

It is estimated that at least 20,000 to 25,000 couples receive in-vitro fertilization (IVF) care each year from abroad [26]. In a 2008 report, the fertility industry was seen as an important growth sector with additional revenue earning potential of $1-2 billion by 2012 [27]. Turkey is ranked 7^th^ in the global IVF market with more than 120 Assisted Reproduction Centers operating in the country. Total number of baby born with IVF in Turkey was 44,000 in 2010 [28].

Many of the IVF patients prefer to seek care from foreign countries for a number of reasons. Prohibitions, restrictions and regulations (e.g. restrictions on surrogacy, donor eggs, donor spermatozoa and age limitations), long waiting line for the service, high out-of-pocket cost and uncertainty about success of the treatment are considered the major reasons. In general, the IVF treatment is such that it rarely exposes the provider to malpractice risk and many providers are willing to participate in this service provision. Since the procedure does not make patients sick or require them to remain in the clinic or hospital for an extended period of time, patients can often combine obtaining IVF services with general tourism.

Although Turkey has become an important IVF medical tourism destination, interestingly, it is not because of its lower restrictions or prohibitions in terms of reproductive choices individuals face. For example, egg donation, surrogacy and sex selection are officially prohibited in Turkey [29]. Therefore, international movement of patients to obtain IVF services from Turkey is probably related to factors like cost, quality and convenience of obtaining the services.

The possibility of adverse consequences on access to health care in the host country has been mentioned in the literature but IVF services should not lower availability of other general medical care services. Medical Tourism for IVF is unlikely to affect availability of medical personnel in other health care services and therefore will have little or no impact on availability of general health services for the population. With more than 120 IVF laboratories in the country, access to IVF services is also not a concern for Turkey. Even though making IVF services to foreign patients may increase the price of this service in the marketplace, it is unlikely to affect the out-of-pocket costs for Turkish patients. In Turkey, first and second cycles of IVF are paid by the governmental social security agency and increased market price may increase pressure on governmental budget but unlikely to affect costs paid by patients.

One potential issue working against continued expansion of medical tourism in Turkey is the political uncertainty and disruptions created by wars and conflicts in the Middle East. It is not clear how the volatile political environment will affect medical tourism. If the armed conflict becomes more widespread with spillover effects in Turkey, the perceived cost of obtaining services from Turkey may exceed the perceived benefits reducing the demand for medical tourism in Turkey.

## Methods

This study has used a Joint Commission International (JCI) accredited, Johns Hopkins Medicine affiliated, ESMO-European Society for Medical Oncology certified hospital in Turkey to better understand the IVF service provision in Turkey and reasons for seeking care from the facility by international patients. The hospital was selected for this case study because of its popularity among medical tourism patients [30]. The hospital has an embryology lab with advanced medical devices. The chief of IVF department of the hospital is an American Board of Obstetrics and Gynecology certified physician with license to provide IVF services in the USA. The IVF Team in this hospital is composed of highly experienced, English speaking members. Although the choice of a highly successful health center will not be representative of all facilities in the country but medical tourism is not based on the “average” facilities in the country. Therefore, better understanding of medical tourism requires focusing on the few high-quality health facilities that cater to the needs of international patients.

Process of IVF treatment adopted by the hospital is as follows. The information was obtained through an interview of IVF service providers in the hospital.

1. The couple is asked to complete some medical tests in home country and to send the test results to the hospital. Based on the test results, doctors evaluate the patients’ eligibility for treatment and potential chance of success for the case. The hospital communicates the initial evaluation by the physicians to the couples.
2. After arriving in the hospital in Turkey, an ultrasound test is done and the patient takes stimulating injections for 10 days. After that the Ovum Pick-up (OPU) is made.
3. 3–5 days after OPU, transfer is done and the couples wait another two days in Turkey for subsequent evaluation and rest.
4. Patient takes the pregnancy test in the home country and sends the results.
5. Doctor informs the patient the medications she should be on till the 9^th^ week of pregnancy. After that the patient’s doctor in her home country becomes the principal health care provider.

In this case study we have compared quality of care, IVF success rate and cost of obtaining the services in the study hospital in Turkey and in the USA. Since this hospital is the leading IVF provider in Turkey, the hospital-specific information should indicate service quality and costs of IVF services for patients from abroad. Another advantage of using this hospital as the case study is that the hospital measures and reports the same JCI required indicators available for the US hospitals. The comparison of IVF service providers in the USA in general with one specific IVF provider in Turkey may appear as biased but from the perspective of patients this type of comparative analysis is more relevant than comparing average values for USA and Turkey. International patients do not consider average quality and cost of health care services in the destination countries. In fact, the average quality of health care services in some of the destination countries, like India, is relatively poor and if international patients considered the average quality, these countries could not have become important exporters of health services.

## Results and discussion

The IVF service should have little or no impact on general clinical quality, often measured by indicators like infection rates. These indicators, however, provide some idea about overall service quality of the selected hospital and the hospital sector in general for USA. Table 1 reports a number of quality indicators for the Turkish hospital for 2011. The indicators for US hospital system are also reported in the table (Table 1).

**Table 1:** General Service quality measures for the Turkish Hospital and the Hospital sector of USA (2011)

Indicator of clinical service quality	Turkish hospital^*^	US hospital sector^*^
Urinary Tract Infections (UTI) (per 1000 catheter days)	2.2	2.4
Catheter related blood stream infections (per 1000 catheter days)	0.7	1.8
Patient Falls (Per 1000 Inpatient Days)	0.6	1.3–8.9
Pressure Ulcer (Per 1000 Inpatient Days)	0.6	5.2
Prophylactic Antibiotic Received Within 1 h Prior to Surgical Incision	99.4	--
Prophylactic Antibiotics Discontinued Within 24 h After Surgery End Time	97.3	--

*Source: the target hospital in Turkey and general quality outcomes for short-stay hospitals in the USA

In the Turkish hospital, urinary tract infection rate was 2.2 compared to 2.4 for major teaching hospitals in the USA. Catheter related blood stream infections per 1000 catheter days was 0.7, less than half the rate for US teaching hospitals [31]. The Agency for Health Care Research and Quality (AHRQ) review of observational studies in acute care hospitals reported that accidental fall ranged from 1.3 to 8.9 falls/1,000 patient days in the USA [32], significantly higher than the rate for the Turkish hospital. The risk-adjusted rate of Pressure Ulcer per 1000 inpatient days was 5.18 in 2008 for community hospitals in the USA as reported by AHRQ [33]. Therefore, the quality of clinical services in the Turkish hospital appears to be better than the teaching hospitals and other community hospitals in the USA. Although this comparison is biased in favor of Turkey, the quality indicators of the target hospital possibly indicate one of the reasons for selecting the health facility by international patients to obtain medical care services.

To understand the cost differentials between USA and Turkey for IVF treatment, this study collected cost information of IVF service package. A number of IVF specific services and cost-items were considered when analyzing the cost. The hospital in Turkey offers a fixed package price for IVF and the package price includes all the relevant services like Monitoring, Ultrasonography examinations, physician consultations; Egg retrieval, anesthesia, IVF; Embryology laboratory services, Assisted hatching, IMSI, Blastocyst, Embryo Transfer; Airport/hospital or hotel/hospital ground transportation; Hotel and trip arrangements and 24/7 translator assistance. These costs are reported in Table 2. Since the costs are based on market prices, these should reflect total value of all the resources used, including the fixed cost items.

**Table 2:** Cost per IVF visit in Turkey in US Dollars (based on the package price offered by IVF service provider)

Cost items	Cost per IVF case in US $
Medical Cost	2500
Airfare (15–20 % Turkish airlines discount for medical tourism patient)	2500
Hotel (located in hospital campus, offered as part of IVF package)	1500
Other expenses (at 100 USD per day)	2000
TOTAL	8500

As Table 2 indicates, total cost related to IVF treatment in Turkey becomes about $8,500. The American Society for Reproductive Medicine reported that the average cost of an IVF cycle in the United States is about $12,400 [34]. Medical tourism web sites list the prices or costs of IVF in different countries, although it is not clear what cost items were included in deriving the total costs. The IVF cost per case ranged from $ 4,500 to 5,700 for other developed countries ($4,523 for Norway; $5,300 for Sweden; $5,504 for Italy; $5,600 for Spain; $5,766 for Canada) (ivfcost.net 2015) but the costs are about $2,180 in Korea, $2,700 in Jordan, $2,800 in Costa Rica, $3,950 in Mexico, and $3,819 in Malaysia (taken from medicaltourism.com). Note that direct medical care cost for IVF in Turkey is about $2,500, a very competitive price even when compared with the prices in other developing countries.

The cost difference between USA and Turkey is so significant that travel and accommodation expenses may not offset the cost-advantage of Turkey. For example, total cost for patients travelling from the USA to Turkey at current levels of prices are: Medical care cost $2,500, round trip airfare for two with 20 % discount offered by Turkish Airlines for medical tourism $2,500, hotel expenses for 10 days $1,500, other expenses $100 per day for 10 days $1000. Total cost of a patient from the USA seeking care in Turkey becomes about $8,500, not adjusting for the opportunity the trip offers for general tourism. If the patient seeking care in Turkey comes from Europe, total cost will be significantly lower. Therefore, the aggregate cost of treatment in Turkey including the travel expenses remains below the average medical care cost in the United States or Europe. In USA, most patients do not have insurance coverage for IVF services. The National Infertility Association in the USA reports that only 15 of US states have laws requiring insurance coverage for infertility treatment [35].

Another important factor in decision-making of patients is the success rate of the procedure in the destination country compared to that in the home country. His paper has used successful pregnancy rate as the measure of effectiveness. Take-home baby rate was not used as the effectiveness measure because proximate measure if success of IVF treatment should be the clinical pregnancy cases. Most patients receiving IVF services internationally leave for their home country after the pregnancy happens. The data on birth of the baby are often not available and take-home baby rate is affected by many other factors which are beyond the control of IVF services. Clinical pregnancy rate has been used as the measure of outcome in this article. Table 3 compares the success rates of IVF in the Turkish hospital and in USA [36]. Note that the success rates, measured by clinical pregnancy rates, of IVF treatment in the Turkish hospital, where most of the IVF patients from abroad are treated, are much better than the average of US success rates in all the age groups excepting the older age group (more than 42 years of age). Even in this age group, the success rate in the Turkish hospital is no worse than the US rate. On the average, the success rate of IVF treatment in Turkish hospital appears to be 50 % higher than that in the USA.

**Table 3:** Success rates of IVF treatment in the USA and in a Turkish Hospital by age group of patients

Age groups of patients	IVF success rates (percentages)	
	Turkish Hospital in the case-study^*^	Average rate for the USA^*^
<35 age	68	48
35–37 years	63	39
38–40 years	44	30
41–42 years	32	20
>42 years	9	9

*Source: data from the most important IVF service provider for foreign patients in Turkey and the averages for IVF providers in USA

Using the clinical pregnancy rate as the effectiveness measure, we can now derive the cost-effectiveness of IVF services in Turkey and in the USA. Table 4 reports the cost-effectiveness of IVF services expressed as cost per successful clinical pregnancy. For the calculations, we have used the average age of patients to define the average success rate in each of the countries. It is assumed that 80 % of the individuals seeking IVF services in Turkey are in the age groups 38 years or higher. Using this proportion, the successful pregnancy rate for Turkey and USA should be about 37.7 and 25.5 % respectively. Using the average cost of $8,500 and $12,400 in these two countries, cost per successful case becomes about $22,500 in Turkey and $48,600 in the USA. If the success rate in Turkey reduces by 10 %, the cost-effectiveness ratio will become $25,000 in Turkey. Changes in the effectiveness and cost parameters by 10 % do not affect the cost-effectiveness ratios of Turkey that much-the worst cost effectiveness ratio becomes $27,600 if cost increases by 10 % and effectiveness declines by 10 %. On the other hand, significant improvement in effectiveness in the USA by about 30 % improves the cost-effectiveness ratio from $48,600 to $35,400. Therefore, allowing all reasonable range of changes in total cost and effectiveness, the cost advantage in Turkey remains at least 50 % better than the US cost-effectiveness.

**Table 4:** Cost effectiveness of IVF Services in Turkey and in the USA with Sensitivity analysis (US dollar Cost per Successful pregnancy)

Assumptions about cost and effectiveness parameters	Turkey			USA		
	Success rate	Cost (USD)	Cost per successful pregnancy	Success rate	Cost (USD)	Cost per successful pregnancy
Average success rate based on patient age and average cost	37.7	8,500	22,546	25.5	12,400	48,627
Reduction in success rate by 10 % in Turkey and improvement in USA (lower average age of patients in the USA)^a^	33.9	8,500	25,074	29.2	12,400	42,466
Increase in cost by 10 % in Turkey and higher success rate in USA^b^	37.7	9,350	24,801	35.0	12,400	35,429
Increase in cost by 10 % and reduction in success rate by 10 % in Turkey	33.9	9,350	27,581	25.5	12,400	48,627

^a^It is assumed that patients are equally distributed among the five age groups of patients in the USA (age groups in Table 3)

^b^It is assumed that only 20 % of patients in the USA are from the age group more than 40 years while it is 50 % for Turkey, the group with relatively low success rate

## Conclusions

Cost of medical services in Turkey is only about 30 to 50 % of the costs in Western Europe and in the USA but the high demand for IVF services in Turkey by international patients is not due to cost-advantage only. Quality indicators of clinical services and success rate of IVF are better in Turkey than in the USA. After correcting the cost per case for the success rate, the cost-advantage of Turkey becomes very high. Since the overall quality indicators of Turkish hospitals (especially accredited ones) have improved significantly over the years, the demand for medical tourism in Turkey is expected to increase in the future.

With the advancement of medical technology, quality of hospital services is becoming increasingly homogenized around the world creating opportunities for attracting foreign patients. Even though modern technology adoption makes the medical care sector relatively more capital intensive, it is still a highly labor-intensive industry. High labor intensity implies that the developing countries will continue to have significant cost advantages over developed or transitional economies in the provision of medical care services. As the medical tourism expands, countries try to define their own areas of specialization to protect market share. It is expected that Turkey will also define its own areas of specializations. One of the medical specialty areas where Turkey appears to have significant cost and quality-advantages is the provision of IVF services. Specializing in specific types of medical interventions or treatments should improve efficiency of the health system and help attract foreign patients to the country.

Another important lesson from this case study is that competitive advantage in global medical tourism market requires not only the presence of high-quality hospitals and medical professionals in the country, it is also important to develop hospitals and medical facilities that provide services mainly to patients from other countries. Dedicated facilities to meet the needs of medical tourists improve confidence in the ability of destination country facilities to address the needs of patients. Just like other developing countries of the world, Turkey is competing with regional hubs to develop its medical tourism industry and any confidence-building step or strategy will help improve the market demand. If the medical facilities providing services to medical tourists can maintain their cost-advantage without sacrificing the quality of care, demand for medical services by patients from outside the country is likely to expand and fertility treatment may be used by policy-makers as the catalyst for future development of the sector.

## Abbreviations

AHRQ, Agency for Health Care Research and Quality; ESMO, European Society for Medical Oncology; IVF, in-vitro fertilization; JCI, Joint Commission International; OECD, Organization of Economic Cooperation and Development; OPU, ovum pick-up
