# Correction to: Opportunities for reproductive tourism: cost and quality advantages of Turkey in the provision of in-vitro Fertilization (IVF) services

**DOI:** 10.1186/s12913-017-2637-x

**Published:** 2017-11-06

**Authors:** M. S. Yildiz, M. Mahmud Khan

**Affiliations:** 1 Internal Audit Department, Turkey Ministry of Health, Ankara, Turkey; 2 Department of Health Services Policy and Management, University of South Carolina, 915 Greene Street, #357, Columbia, SC 29208 USA; 3 Department of Health Services Administration, China Medical University, Taichung City, Taiwan

## Correction

After the publication of this article [1] we were made aware that the affiliation for the first author was shown incorrectly as “Ministry of Health, Ankara, Turkey”. The correct affiliation is “Internal Audit Department, Turkey Ministry of Health, Ankara, Turkey” and has been included in this correction.

## References

[1] Yildiz MS, Khan MM (2016). Opportunities for reproductive tourism: cost and quality advantages of Turkey in the provision of in-vitro Fertilization (IVF) services. BMC Health Serv Res.

